# Retraction: Nexus between energy efficiency, green investment, urbanization and environmental quality: Evidence from MENA region

**DOI:** 10.1371/journal.pone.0323298

**Published:** 2025-05-07

**Authors:** The PLOS One Editors

The *PLOS One* Editors retract this article [1] due to concerns about potential manipulation of the publication process. These concerns call into question the validity and provenance of the reported results. We regret that the issues were not identified prior to the article’s publication.

MQ did not agree with the retraction. CG and HS either did not respond directly or could not be reached.
